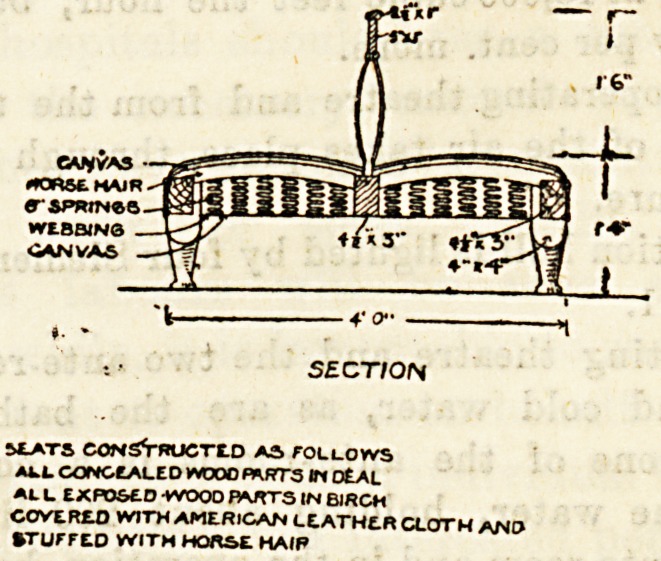# Gloucester Asylum

**Published:** 1892-04-09

**Authors:** 


### FURNITURE AND FITTINGS.

### GLOUCESTER ASYLUM.

It is quite remarkable how enormous have been the strides in the direction of increased comfort and completeness of detail in the internal arrangements of public asylums in this country, and especially in the county asylums. Nothing that can be done to promote one or the other seems absent nowadays. The taste displayed by many of the Medical Superintendents of our English Asylums in the decoration of the wards, the day rooms and corridors, as well as the recreation halls, is so admirable as to afford an object lesson to the public, who might with advantage take a hint from many asylums for the improvement of their own houses. Owing to the stricter classification and grading of the insane, according to their mental condition and habits, the harmless and inoffensive are now provided in most of the county asylums with really charming day rooms which have an air of brightness and comfort that cannot fail to have a beneficial effect upon the occupants. At the new Gloucester asylum the whole of the heating, cooking, and baking is done by water gas, the fittings having been supplied in the kitchens by the well-known firm of Slater and Co., of High Holborn. Unfortunately, either through want of experience or by an oversight, the whole of the coppers are made of copper metal, which causes the boiler portion of the kitchens and also the hot water system to be in practice a failure. The action of the water-gas on the copper is so deleterious, that after three months' wear the coppers have to be taken out and repaired, or renewed. The cost of the gas is absurdly small, being only 2½d. per thousand feet. If the gas companies can manufacture gas at this rate, the charge of two shillings and sixpence or three shillings per thousand feet, to say nothing of six or seven shillings, which is sometimes the charge in small communities, would seem to justify the persistent outcry on the part of the consumers.

We were much interested to notice at the New Gloucester Asylum that all the bedsteads are of wood. These bedsteads are so constructed as to leave no corners or points to which a patient with suicidal instincts can fasten anything. Instances prove that suicidal patients are able, where iron bedsteads are used in single rooms, to do themselves serious injury, and cases have occurred where the patient has attached a loop to the bedstead, and has succeeded in strangling himself. Hence the introduction of these wooden beds at Gloucester, which experience proves are capable of being kept perfectly clean, and of being in all respects preferable to those made of iron.

At the new Gloucester Asylum they have an excellent double couch in the day rooms capable of holding twelve women patients comfortably. It would be improved by the rounding of the head-rail, but in all other respects this piece of furniture may be regarded as a model, and would be well adapted for the purpose of large hospital wards. We give an illustration of this couch with the improvements suggested, and would commend it to asylum superintendents and hospital committees.

Another feature of the Gloucester Asylum may be usefully notified. All the fittings are of pitch-pine, and, instead of the usual painted dado in the corridors, day rooms, and dormitories, a pitch-pine one has been fixed, which adds greatly to the lightness and attractiveness of the whole place.

There is one feature which we are surprised that a representative body like the County Council should tolerate in an asylum. The airing-grounds, instead of being made with sunk fences and low walls, are surrounded by iron railings of a cheap pattern about ten feet high. As the visitor approaches the asylum during the time the patients are taking exercise, the airing grounds remind one of the Zoological Gardens, and anything more melancholy or, on the whole, more objectionable, it is impossible to conceive. No doubt the Gloucester County Council will materially alter this when the asylum is finished. Meanwhile these airing-grounds leave much to be desired, and fill the visitor with feelings of sadness and repulsion. We congratulate Dr. Craddock and Dr. Henley, however, on the general efficiency and excellence of the administrative details.

ELEVATION

SECTION

SEATS CONSTRUCTED AS FOLLOWS

ALL CONCEALED WOOD PARTS IN DEAL

ALL EXPOSED WOOD PARTS IN BIRCH

COVERED WITH AMERICAN LEATHER CLOTH AND

STUFFED WITH HORSE HAIR

## Figures and Tables

**Figure f1:**
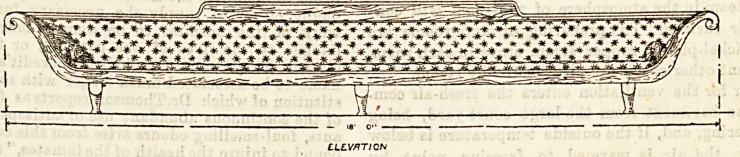


**Figure f2:**
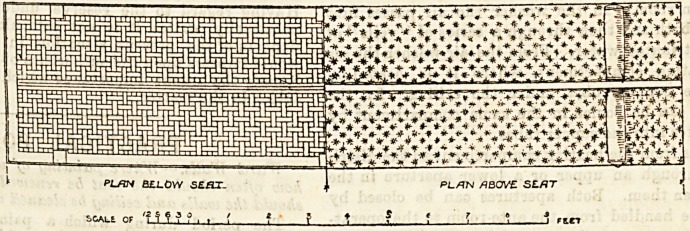


**Figure f3:**